# Interferon Lambda: A New Sword in Cancer Immunotherapy

**DOI:** 10.1155/2011/349575

**Published:** 2011-12-06

**Authors:** Ahmed Lasfar, Walid Abushahba, Murugabaskar Balan, Karine A. Cohen-Solal

**Affiliations:** ^1^Department of Biochemistry and Molecular Biology, University of Medicine and Dentistry of New Jersey-New Jersey Medical School, University Hospital Cancer Center, 205 South Orange Avenue, Newark, NJ 07103, USA; ^2^Department of Medicine, Division of Medical Oncology, UMDNJ-Robert Wood Johnson Medical School, The Cancer Institute of New Jersey, 195 Little Albany Street, New Brunswick, NJ 08903, USA

## Abstract

The discovery of the interferon-lambda (IFN-**λ**) family has considerably contributed to our understanding of the role of interferon not only in viral infections but also in cancer. IFN-**λ** proteins belong to the new type III IFN group. Type III IFN is structurally similar to type II IFN (IFN-**γ**) but functionally identical to type I IFN (IFN-**α**/**β**). However, in contrast to type I or type II IFNs, the response to type III IFN is highly cell-type specific. Only epithelial-like cells and to a lesser extent some immune cells respond to IFN-**λ**. This particular pattern of response is controlled by the differential expression of the IFN-**λ** receptor, which, in contrast to IFN-**α**, should result in limited side effects in patients. Recently, we and other groups have shown in several animal models a potent antitumor role of IFN-**λ** that will open a new challenging era for the current IFN therapy.

## 1. Introduction

**Figure 1:**
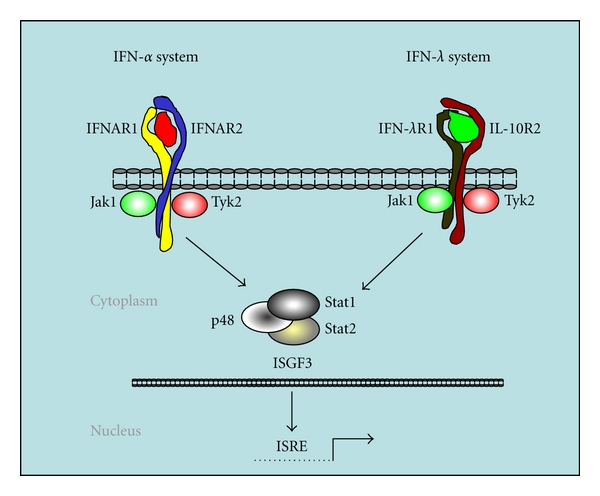
IFN-*α* and IFN-*λ* receptor systems and cell signaling. IFN-*α* and IFN-*λ* interact with distinct receptors, but the downstream signaling is similar. IFN-*α* interacts with receptors composed of IFNAR1 and IFNAR2, and IFN-*λ* interacts with a receptor composed of a specific chain, IFN-*λ*R1, and IL-10R2, a shared subunit with IL-10, IL-22, and IL-26. Both IFNs lead to the activation of the Jak kinases (Jak1 and Tyk2) and the formation of the transcription-complex-designated IFN-stimulated gene factor 3 (ISGF3), which includes p48, Stat1, and Stat2. ISGF3 complex binds to the IFN-stimulated response element (ISRE) and induces gene transcription.

Despite the early discovery of interferon (IFN) in 1957, IFN lambdas were just identified during the recent years and classified as a new group, type III IFN. In human, 3 distinct proteins called IFN-*λ*1, IFN-*λ*2, and IFN-*λ*3 have been identified [1, 2]. They are also named interleukin-29 (IL-29), IL-28A, and IL-28B, respectively [3]. The members of this new IFN family were found to interact through unique receptors that are distinct from type I (IFN-*α*/*β*) and type II (IFN-*γ*) IFN receptors. The receptor for type III IFN is composed of the unique IFN-*λ*R1 chain also called IL-28AR and the IL-10R2 chain, which is shared with IL-10, IL-22, and IL-26 receptor complexes. Although type III IFNs bind to a specific receptor, the downstream signaling is similar to that induced by type I IFNs. Both type I and type III IFNs stimulate common signaling pathways, consisting of the activation of Jak1 and Tyk2 kinases and leading to the activation of IFN-stimulated gene factor 3 (ISGF3) transcription complex. ISGF3 is composed of STAT1 and STAT2 and the interferon regulatory factor IRF9 (ISGF3-*γ* or p48) (Figure 1). Although there are three genes encoding highly homologous but distinct human IFN-*λ* proteins (IFN-*λ*1, IFN-*λ*2, and IFN-*λ*3), our search of the mouse genome revealed the existence of only two genes, representing mouse *IFN-*λ*2* and *IFN-*λ*3* gene orthologues, located in chromosome 7 and encoding intact proteins. The mouse *IFN-*λ*1* gene orthologue is a pseudogene containing some variations in addition to a stop codon in the first exon and does not code for an active protein [4]. We have cloned the mouse IFN-*λ*s (mIFN-*λ*2 and mIFN-*λ*3) and IFN-*λ* receptor (mIFN-*λ*R1) orthologues and found them to be quite similar to their human counterparts. Experiments showed that similar to their human counterparts, mIFN-*λ*2 and mIFN-*λ*3 signal through the IFN-*λ* receptor complex, activate ISGF3, and are capable of inducing antiviral protection and MHC class I antigen expression in several cell types. The results showed that murine type III IFNs (IFN-*λ*s) engage a unique receptor complex, composed of IFN-*λ*R1 and IL-10R2 subunits, to induce signaling and biological activities similar to those of type I IFNs. Interestingly, in contrast to type I and type II IFNs, type III IFNs demonstrate less species specificity.

## 2. Biological Properties of IFN-***λ***

### 2.1. Restrictive Cell Response to Type III IFN (IFN-*λ*s)

**Figure 2:**
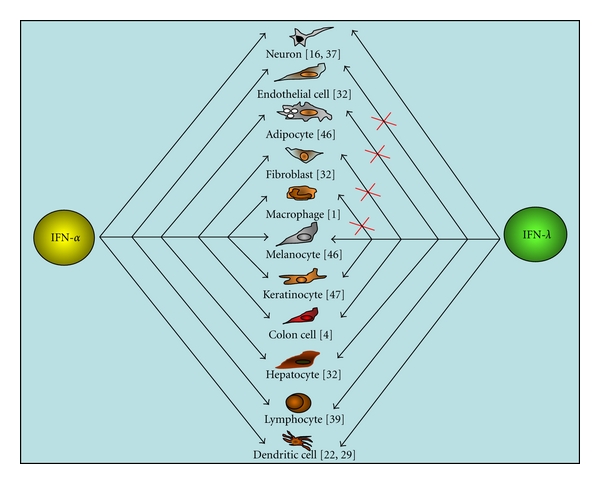
Cellular targets for type I and type III IFNs. Response to IFN-*α* and IFN-*λ* in cells from different origins in human. The IFN response was assessed by measuring the IFN-induced cell signaling (Stat activation) and cell activity (MHC class I antigen stimulation). In contrast to IFN-*α*, only restricted cells respond to IFN-*λ*, including epithelial-like cells, forming the major organs of the body.

Although type I and type III induced similar cell signaling, the intensity of cell signaling as measured by STAT1 activation appeared to be significantly lower for type III IFNs [4]. In comparison with type I IFN, only restricted cell types respond to type III IFN (Figure 2). Interestingly, we did not find a strict correlation between the intensity of cell signaling induced by IFN-*λ* and the level of biological activity. For example, in B16 melanoma cells, although IFN-*λ* induced a very weak STAT1 activation in comparison with IFN-*α*, we observed a robust stimulation of MHC class I expression at the cell surface, indicating the potential contribution of cell-specific modulators of the IFN-*λ* activity.

Antiviral studies performed *in vitro* and *in vivo* have shown that both IFN-*α* and IFN-*λ* contribute to the overall host antiviral defense system [2, 3, 5–8]. It has been demonstrated that IFN-*λ* induces antiviral activity against VSV (vesicular stomatitis virus) and EMCV (encephalomyocarditis) in many human cell lines [2, 3, 9, 10]. However, by using different mouse models of viral infection, Ank et al. demonstrated that IFN-*λ* was effective against DNA virus, simplex virus 2 HSV2 but not RNA viruses such as EMCV and lymphocytic choriomeningitis virus LCMV [6]. Several other studies demonstrated that type III IFNs can also inhibit replication of hepatitis C virus (HCV) and hepatitis B virus (HBV) *in vitro* [10–14]. These studies were important since they underlined the fact that IFN-*λ* could be used as an alternative to IFN-*α* for HCV patients who are resistant to IFN-*α* treatment. It has been reported that IFN-*λ* has the ability to inhibit human immunodeficiency virus type 1 (HIV-1) infection of blood monocyte-derived macrophages that expressed IFN-*λ* receptors [15] and the herpes simplex virus type 1 (HSV1) infection of human astrocytes and neurons [16]. However, in most other cases, the antiviral potency of IFN-*λ* against several viruses seems to be lower than that of IFN-*α* [2, 3, 8, 9, 13, 17]. In addition, IFN-*λ* and IFN-*α* may induce distinct signal transduction and gene regulation kinetics [13, 18].

Moreover, type I IFN-*α* activates a plethora of innate and adaptive immune mechanisms that help eliminate tumors and viral infections. IFN-*α* immunoregulatory functions include major histocompatibility complex (MHC) class I expression in normal and tumor cells, activation of NK cells, dendritic cells (DCs), and macrophages, resulting in the promotion of adaptive immune responses against tumors and virally infected cells [19, 20]. The role of IFN-*λ* in the immune system is currently being investigated by several groups. So far, data suggests that IFN-*λ* exerts immunomodulatory effects that overlap those of type I IFN. It has been recently demonstrated that human IFN-*λ*1 (IL-29) modulates the human plasmacytoid DCs function and cytokine response [21, 22]. IFN-*λ*1 treatment of whole peripheral blood mononuclear cells (PBMCs) upregulated the expression of IL-6, IL-8, and IL-10 but not IL-1 or TNF. This IFN-*λ*-induced cytokine production was inhibited by IL-10. By examination of purified cell populations, it was also shown that IFN-*λ*1 activated monocytes, rather than lymphocytes, resulting in the secretion of the above panel of cytokines, suggesting that IFN-*λ*1 may be an important activator of innate immune responses particularly at the site of viral infections [21]. IFN-*λ*1 was also shown to possess immunoregulatory functions on T helper 2 (Th2) responses by markedly inhibiting IL-13. However, only moderate effect was observed on IL-4 and IL-15, the other important cytokines in the Th2 response [23–25]. This immunoregulatory function was enhanced through the expression of IFN-*λ*R1 on CD4^+^ T cells [23]. These findings correlate with data suggesting that IFN-*λ* may have an immunoprotective role against asthma, the allergy disease caused by an exaggerated Th2 response [9, 26, 27].

Similar to IFN-*α*, IFN-*λ* produced by DCs, in response to toll-like receptor (TLR) stimulation, was found to have specific effects on DC differentiation and maturation [28], which include only partial maturation of DCs, upregulation of MHC class I and II molecules, and no induction of co-stimulatory molecules [9, 29]. During their differentiation from monocytes, DCs acquire IFN-*λ* responsiveness through the expression of IFN-*λ*R1. Interestingly, DCs treated with IFN-*λ* promoted the generation of tolerogenic DCs and the IL-2-dependent proliferation of Foxp3-expressing CD4^+^CD25^+^ regulatory T cells (Tregs) [29]. More recently, Morrow et al. have demonstrated, through DNA vaccination with plasmids encoding IFN-*λ*3 (IL-28B) and IL-12, that IFN-*λ*3, just like IL-12, is able to enhance adaptive immunity. However, in contrast to IL-12, IFN-*λ*3 reduces regulatory T-cell populations. They also showed that unlike IL-12, IFN-*λ*3 is able to increase the percentage of splenic CD8^+^ T cells in vaccinated animals and that IFN-*λ*3 can completely protect mice from death following a lethal influenza challenge [30]. These studies altogether highlight the strong candidacy of IFN-*λ* as a potential novel immunotherapeutic agent.

In addition to antiviral and immunomodulatory activities, type I IFNs demonstrate antiproliferative activities in most cell lines, while this activity seems to be restricted with IFN-*λ*s [9, 17]. Type I IFNs have been shown to induce apoptosis in tumor cells, yet the molecular mechanisms mediating cell death in response to these IFNs remain to be fully explained. By binding to their corresponding cellular receptor complexes, IFNs induce a quick and potent signaling which leads to the expression of more than 300 IFN-stimulated genes (ISGs) [13, 31, 32]. Many ISGs encode proteins that have been implicated in apoptosis [33, 34]. Unlike IFN-*α*, IFN-*λ*s do not inhibit the proliferation of several cell lines including the Daudi cells (a B-lymphoblastoid cell line from Burkitt's lymphoma), which strongly respond to type I IFNs in an antiproliferative assay [2, 3, 10, 17]. However, it was demonstrated that IFN-*λ*s do inhibit the proliferation of few tumor cell lines, such as the LN319 human gliobastoma cell line [17] and of cells constitutively expressing high levels of IFN-*λ*R1 [35]. The antiproliferative effects of IFN-*λ* have been demonstrated in various tumor cell lines that express ectopic or endogenous IFN-*λ* receptors [17, 36, 37]. Therefore, the ability of IFN-*λ*s to induce antiproliferative activity in cells depends on the level of IFN-*λ*R1 expression.

It has been recently reported that IFN-*λ* signaling in colorectal adenocarcinoma HT29 cells led to caspase activation, externalization of phosphatidylserine (PS), and DNA fragmentation, resulting in subsequent apoptosis [38]. This study provided evidence for the first time that type III IFNs, alone or in combination with other stimuli, have the potential to induce apoptosis. Moreover, another recent study revealed that IFN-*α* and IFN-*λ* differ in their antiproliferative effects and this was correlated with a difference in the duration of JAK/STAT signaling activity between the two IFNs and prolonged ISG expression upon IFN-*λ* treatment [18]. Using the human keratinocyte HaCaT cell line that expresses receptors for both IFN-*α* and IFN-*λ*, they found that IFN-*λ* induced a more pronounced growth inhibitory effect than IFN-*α*. IFN-*λ* was also more efficient than IFN-*α* in inducing an antiproliferative effect that overlapped with the activation of apoptosis. Prolonged duration of IFN-*λ*-induced STAT activation, and ISG expression could account for the enhanced antiproliferative and proapoptotic effects observed in HaCaT cells, effects not seen upon treatment with high doses of IFN-*α* [18]. Interestingly, a study has shown that IFN-*λ* can induce the growth of human multiple-myeloma cells and antagonize the dexamethasone-induced cell death in these cells [39]. IFN-*λ*-mediated cell growth of multiple myeloma cells was MAPK dependent [39]. High level of IFN-*λ* was found in the malignant bone marrow microenvironment, implying that IFN-*λ* may play a direct role in multiple myeloma development.

### 2.2. Tissue and Species Specificity of Type III IFN (IFN-*λ*)

By using a plasmid electrotransfer approach, Sommereyns and coworkers reported a differential response to IFN-*λ* in mice, with a very low response to IFN-*λ* for the liver, central nervous system, and spleen. However, a high response to IFN-*λ* was observed in the stomach, intestine, heart, kidney, and lung [40]. The IFN-*λ* response was restricted to epithelial cells and correlated with the expression of IFN-*λ*R (IL-28Ralpha). Paradoxically in mice, in spite of the epithelial nature of the hepatocytes, the liver expressed low levels of IL-28Ralpha and responded poorly to IFN-*λ* [8, 40]. However, a significant response to IFN-*λ* was reported in human hepatocytes [13, 32], suggesting the existence of some variations in the response to IFN-*λ* between mice and humans, at least in the liver. Although the main IFN-*λ* targets are the epithelial cells, the presence of potential tissue-specific factors may modulate the IFN-*λ* response through the IFN-*λ* receptors. Recently, it has been shown in mice that in contrast to the hepatocytes, prominent response to IFN-*λ* was observed in intestinal epithelial cells. In comparison with IFN-*α*, this response is higher and plays a critical role in protecting the intestinal epithelium from viral infection [41], strongly suggesting the prominent role of IFN-*λ* in organs with mucosal surface at least in mice [6, 42, 43]. In addition to the direct effect of IFN-*λ* on the mucosal epithelium, local immunomodulations can also be promoted [44].

### 2.3. Distribution of IFN-*λ*R1 and Responsiveness to IFN-*λ*

The functional IFN-*λ*R is formed by two chain proteins, IFN-*λ*R1 (also called IL-28Ralpha) and IL-10R2. IFN-*λ*R1 is unique for the IFN-*λ*s, and its tissue distribution is highly restricted. In contrast to IFN-*λ*R1, IL-10R2 is shared by IL-10, IL-22 and IL-26 and ubiquitously expressed in all tissues. Unlike IFN-*α*, only few cell types respond to IFN-*λ* (Figure 2). In contrast to the epithelial-like cells, fibroblasts and endothelial cells were completely unresponsive to IFN-*λ* [4]. Although the hematopoeitic system is not the primary target of IFN-*λ*, the response of some subpopulations to IFN-*λ* is not excluded. In mice, we found that IFN-*λ* induces STAT1 activation in both plasmacytoid and myeloid dendritic cells [45]. These results are in accordance with those obtained by Mennechet and Uzé [29], who proposed the acquisition of an IFN-*λ* response by monocytes after their differentiation into dendritic cells. Therefore, the response to IFN-*λ* may be controlled by the induction of the IFN-*λ*R1 expression. Different levels of IFN-*λ*R1 were found in different tissues [40, 43, 46]. The highest levels were found in the gastrointestinal tract and lung. The brain showed the lowest level of receptor expression. The IFN-*λ*R1 expression was also analyzed in different cell types. The expression of cell populations isolated from human skin showed a high expression of IFN-*λ*R1 in keratinocytes and melanocytes. However, dermal fibroblasts, endothelial cells, and subdermal adipocytes did not express significant amounts of IFN-*λ*R1. Significant expression of IFN-*λ*R1 was detected in primary human hepatocytes in comparison with the chondrocytes, isolated from the hyaline cartilage of the knee joint [46, 47]. Although the expression of IFN-*λ*R1 was significantly high in lymphoid tissues, the IFN-*λ* response was very weak, implying the presence of specific mechanisms in the lymphoid tissues that may inhibit the IFN-*λ* response. For example, IFN-*λ*R1 levels in B cells are threefold those detected in keratinocytes, which exhibit one of the highest responses to IFN-*λ*. Witte et al. proposed the potential role of soluble IFN-*λ*R1, highly released by the immune cells, in this weak response to IFN-*λ* [46].

Although all the IFN-*λ*s interact with the same receptor, IFN-*λ*R1, the binding characteristics for each ligand are still under investigation. In the future, it will be important to analyze the IFN-*λ* activity in light of the IFN-*λ* binding to the cells and understand particularly the role of IFN-*λ*3, which possesses the highest activity as compared with the other IFN-*λ*s [48, 49]. Analysis of the ligand binding in combination with the activity induced by IFN-*λ* will be also important in understanding the impact of IFN-*λ* in epithelial cells, particularly in comparison with the immune cells expressing IFN-*λ*R1. Besides several carcinomas, originating from epithelial cells, which respond to IFN-*λ*, other tumors not arising from epithelial cells may become more sensitive to IFN-*λ*. It was reported that multiple myeloma cells, which originate from B-cell plasmocytes, showed high binding and response to IFN-*λ* [39]. Studying the IFN-*λ* binding in transformed cells versus normal cells may be very helpful for tumor targeting and for the establishment of the optimum dose of IFN-*λ* to be used for the *in vivo* treatment. IFN-*λ* can also be used as a drug carrier, to specifically target a drug to tumors expressing high IFN-*λ* binding sites.

### 2.4. Antiviral Protection in IFN-Type-III-Deficient Mice

The availability of IFN-*λ*R1 knock-out mice allowed for the investigation of the role of type III IFNs *in vivo*. By using those mice, Mordstein et al. showed for the first time the contribution of IFN-*λ* in the innate immunity against the influenza virus [8]. Later, they found that IFN-*λ* played an important role in the defense against other pathogens that infect the respiratory tract, such as the respiratory syncitial virus, the metapneumovirus, and the severe acute respiratory syndrome (SARS) coronavirus. However, the lassa fever virus which replicates in the liver, was not affected by the lack of IFN-*λ*R1 [50]. Although this study clearly demonstrated that IFN-*λ* played an important role in protecting the respiratory and gastrointestinal tracts against virus infection, in comparison with type I IFN, the protection provided by type III IFN remains limited. However, in combination, type I and type III may provide a better viral protection. When the response to both type I and type III is deficient, the mice are not able to clear the SARS coronavirus from the intestine as compared with mice in which type I or type III remains functional, implying that IFN-*λ* may strengthen the antiviral activity by acting as a first line of defense for the mucosa [8, 50].

### 2.5. Clinical Use of Type III IFN

The first use of IFN-*λ* in the clinic has started for hepatitis C. The phase 1b study has been conducted in patients with chronic genotype 1 hepatitis C virus infection ((HCV) [51]). Pegylated IFN-*λ*1 in combination or not with ribavirin (RBV, which belongs to a class of antiviral medications called the nucleoside analogues) has been used in this study to assess the efficacy and the potential cytotoxicity. The study was performed in 3 parts. The first part evaluated the pegylated IFN-*λ* as single agent for relapsed patients after IFN-*α*-based treatment. The second part concerned the combination of pegylated IFN-*λ* and RBV in treatment-relapse patients. The third part evaluated pegylated IFN-*λ* in combination with RBV in treatment-naïve patients. In addition, different doses (from 0.5 to 3 microg/kg) of pegylated IFN-*λ* were used. Fifty-six patients were enrolled. 24, 25, and 7 patients were used, respectively, for part 1 to 3. The data showed an antiviral activity in all doses of pegylated IFN-*λ* tested. 29% of treatment-naïve patients achieved rapid antiviral response. As expected, due to the limited IFN-*λ*R1 distribution, the treatment was well tolerated with few adverse effects. Minimal flu-like symptoms and limited hematologic suppression were reported. In summary, the authors concluded that weekly pegylated-IFN-*λ* with or without daily RBV for 4 weeks is associated with a clear antiviral activity in patients with chronic HCV. However, this study lacks a direct comparison between IFN-*λ* and IFN-*α* and the influence of viral and patient genotypes. Now it is well accepted that the response to IFN-*α* or the natural clearance of HCV infection is depending on single-nucleotide polymorphisms (SNPs), upstream of IFN-*λ*3, which could be used as biomarkers to help determine the treatment outcome [52]. The first genome-wide association studies (GWAS) in HCV infection were reported by Ge et al. They evaluated the treatment outcome in a group of 1671 patients of mixed ethnicity, receiving pegylated IFN-*α* and ribavirin. An association was discovered between sustained viral response (SVR) to treatment and a cluster of seven SNPs linked to the IFN-*λ*3 gene, with the most significant SNP (rs12979860) demonstrating high statistical significance [53]. Many other studies have replicated these findings, demonstrating the high link between IFN-*λ*3 and treatment outcome [54–61]. However the mechanisms explaining this link remain to be determined. It is not clear yet if this SNP is associated with a constitutive production of IFN-*λ* that may play a role in HCV clearance and the success of IFN-*α* treatment. These results also suggest the therapeutic potential of the IFN-*α* and IFN-*λ* combination therapy as demonstrated for the hepatocellular carcinoma (HCC) mouse model [62].

## 3. Emergence of IFN-***λ*** as a New Antitumor Agent

### 3.1. Characterization of the IFN-*λ* System and Demonstration of Its Antitumor Activity in a Melanoma Model

Although they engage distinct receptors, IFN-*α* and IFN-*λ* induce similar cell signaling (Figure 1). Since IFN-*α* is widely used in the clinic to treat cancer (Table 1), we have investigated the potential antitumor activity of IFN-*λ* by using the mouse B16 melanoma model. We have chosen this cancer model because melanoma is a very aggressive cancer, and one of the therapeutic agents frequently used in the treatment of melanoma is IFN-*α*. Significant improvements in relapse-free and overall survival, with postoperative adjuvant IFN-*α* therapy, have been reported by large and randomized studies [63–65]. However, the beneficial effect of IFN-*α* was only obtained when the patients received high doses (20 MIU/m2 intravenously five times per week). Studies with low doses of IFN-*α* have not shown significant increase in overall survival [66, 67]. Usually, the dose for optimal antitumor activity is higher than the maximally tolerated dose. This dose dilemma profoundly affects the acceptance of IFN-*α* treatment by both the clinicians and the patients. The adverse effects associated with high doses of IFN-*α* include myelosuppression and nervous system disorders. These effects often compromise the beneficial antitumor effect, with premature discontinuation of the treatment or the reduction of the dose of IFN-*α*. Since virtually all the cells of the body respond to IFN-*α*, it is not surprising that the patients develop numerous side effects. Making a dissection between the beneficial and harmful effects of IFN-*α* is a very challenging task, which requires more investigation of the interferon system. To investigate the antitumor effect of IFN-*λ* in melanoma, we have used a gene therapy approach, consisting on the delivery of the IFN-*λ* gene to tumor cells. Gene transfer into tumor cells is very useful approach to test the effectiveness of cytokines in animal cancer models. This approach does not require production and purification of the protein. The secretion of constant amounts of various cytokines by transduced tumor cells at the site of tumor growth could elicit more effective antitumor responses by acting directly on the tumor microenvironment. Another advantage of the cytokine gene transfer into tumor cells versus systemic administration is the potential of inducing the antitumor effect without eliciting the side effects associated with the systemic administration of high doses of cytokines.

To investigate the potential antitumoral role of IFN-*λ*, we first evaluated the response of B16 melanoma cells to IFN-*λ*, by analyzing STAT1 activation and MHC class I antigen expression. In comparison with IFN-*α*, we have found that IFN-*λ* induces weak STAT1 phosphorylation but strong stimulation of MHC class I antigen expression, indicating a difference between IFN-*α* and IFN-*λ* in the link intensity of cell signaling/biological activity. This result warrants further investigation in comparing the response to IFN-*α* and IFN-*λ*. By using gene transfer, we engineered B16 cells, which constitutively produced mIFN-*λ* (B16.IFN-*λ* cells). In response to their secretion of IFN-*λ*, B16.IFN-*λ* cells exhibited constitutively high levels of MHC class I antigen expression. All the C57BL/6 syngeneic mice injected with parental B16 cells developed tumors. However, the constitutive production of mIFN-*λ* by B16.IFN-*λ* cells markedly affected tumorigenicity of the cells. B16.IFN-*λ* cells were either rejected by the host or grew at a slower rate than control parental B16 cells. The antitumor effect of IFN-*λ* was dose dependent. B16.IFN-*λ* cells also inhibited the growth of parental B16 cells when both cell types were injected together [4]. We also used the engineered B16.IFN-*λ* Res. cells, which, in addition to their constitutive IFN-*λ* secretion, are completely resistant to IFN-*λ*, as demonstrated by the lack of IFN-*λ*-induced MHC class I antigen expression. Interestingly, similar to B16.IFN-*λ* cells, we have found a reduction of the tumorigenicity of B16.IFN-*λ* Res. cells, implying the involvement of host antitumor mechanisms induced by IFN-*λ* [4].

Following our report on the characterization of the mouse IFN-*λ* system and the potent antitumor activity of IFN-*λ* in the B16 mouse melanoma model, independent groups confirmed the role of IFN-*λ* as an antitumoral agent in melanoma and other tumor models. To demonstrate the antitumor activity of IFN-*λ*, Sato et al. [68] used the mouse melanoma B16F0 and B16F10 and the Colon26 cell lines transfected with IFN-*λ*2 cDNA. The IFN-*λ*-transduced B16F0 cells showed an increased activity of caspase 3/7, an induction of p21 and a dephosphoryation of Rb, which triggered a cell cycle arrest and apoptosis. These events, obtained, *in vitro*, were apparently associated with a growth delay, observed *in vivo* after the injection of the B16F0 transduced with IFN-*λ*. A delay in tumor growth was also observed after the administration of the Colon26 cells transduced with IFN-*λ*. By using the B16F10 cell line, which represents metastatic mouse melanoma cells, the authors showed that the overexpression of IFN-*λ* significantly inhibited lung metastasis. In another study, to evaluate the antitumor activity of IFN-*λ*, Numasaki et al. [69] first transduced the mouse fibrosarcoma cells, MCA2005, with the retroviral vector PA317IL-28 (IFN-*λ*2). Following the injection of the engineered tumor cells to mice, the authors observed a significant antitumor and antimetastatic effect in mice inoculated with the MCA2005IL-28 in comparison with those injected with the parental tumor cells.

### 3.2. Investigation of the Antitumor Activities of IFN-*λ* in the BNL Mouse Model of Hepatocellular Carcinoma (HCC)

HCC is the most prevalent type of liver cancer. It is the fifth most common solid tumor and the third leading cause of cancer-related death worldwide. It is also the second most lethal cancer with the five-year survival rate below 9% [70–72]. Treatment options for HCC are limited mainly because of the inefficiency of existing anticancer chemotherapeutic drugs against HCC. Unfortunately, due to a lack of biomarkers and screening for HCC, most patients are diagnosed at advanced stages of the disease and do not meet strict selection criteria for potentially curative surgical tumor resection or orthotopic liver transplantation (OLT) [73–75]. In patients with unresectable HCC and preserved liver function, transarterial chemoembolization (TACE) has been shown to prolong survival. However TACE is rarely curative, and progression-free survival beyond 24 months is not frequent [71, 76]. For patients with advanced disease, systemic chemotherapy is of limited benefit because of the resistance of HCC to existing anticancer drugs and the fact that about 50% of patients with HCC die secondary to liver failure from cirrhosis [77, 78]. HCC occurs most frequently in patients with cirrhosis as a result of chronic HBV (hepatitis B virus) and HCV (hepatitis C virus) infections, and alcohol abuse [72, 79]. Although the link between the cancer and the viral infection is not fully understood yet, there is some suggestion that viral infection interferes with signal transduction and consequently disrupts the normal, controlled growth of cells.

Since IFN-*α* is used in the clinic for the treatment of chronic HCV and HBV infections, several studies evaluated the effect of IFN treatment on the incidence of HCC [72]. It was previously shown that the systemic administration of high doses and long-term IFN-*α* into nude mice bearing human HCC with high metastatic potential, following curative resection, inhibited tumor metastatis and recurrence [80]. The majority of clinical studies also concluded that IFN therapy, alone or in combination with ribavirin, decreased the incidence of HCC, particularly in patients with sustained virological response [81–84]. Therefore, IFN alone or, perhaps, in combination with other drugs can be used as a preventive therapy against the development of HCC in HCV- and HBV-infected patients. However, numerous side effects limit the overall tolerability of IFN-*α*, particularly in patients with cirrhosis [85–87].

In the following part of this section, we describe our findings on the antitumor properties of IFN-*λ* in the BNL mouse model of HCC. To evaluate the antitumor activities of both IFN-*λ* and IFN-*α*, we used a gene therapy approach as previously described [4]. We expressed IFN-*λ* and IFN-*α* genes under a strong constitutive promoter in BNL cells and selected stable cell lines, BNL-IFN-*λ* and BNL-IFN-*α*, constitutively expressing IFN-*λ* and IFN-*α* [45]. Since the constitutive expression of IFN-*λ* at the tumor site was found to affect the tumorigenicity of B16 melanoma cells *in vivo *[4], we examined whether similar effects of IFN-*λ* would be displayed in the case of BNL hepatoma. Mice injected with BNL vector or parental BNL cells developed tumors in 4 to 6 weeks, whereas the tumor appearance for BNL-IFN-*λ* cells was significantly delayed. Similar effects were obtained in mice inoculated with BNL-IFN-*α* cells. These experiments demonstrated that constitutive expression of IFNs at the tumor site resulted in the delay of tumor growth *in vivo*. Interestingly, we found that IFN-*α* and IFN-*λ* exhibited similar antitumor activities [45].

## 4. Potential Antitumor Mechanisms of IFN-**α**  and IFN-***λ***

### 4.1. Antitumor Mechanisms of IFN-*α*

Despite the antiproliferative effects of IFN-*α*, it seems that the direct effects on tumor cells may not be the major mechanism by which IFN-*α* displays its antitumor activity. IFN-*α* can act indirectly on the tumor by inhibiting angiogenesis which is induced by the tumors and is required to promote their growth and metastasis [88]. In mice bearing human tumors, it was clearly demonstrated that the antitumor activity of IFN-*α* is associated with the inhibition of tumor angiogenesis in bladder carcinoma [89] and prostate cancer [90]. The involvement of the immune system in the antitumor mechanism of IFN-*α* was strongly suggested by Gresser et al. [91, 92]. Early studies in tumor models have shown that an intact immune system was essential in IFN-*α*-induced antitumor activities. The inhibition of Friend leukemia cells (FLC) by IFN-*α* in mice was shown to depend on the activation of host cells, such as NK cells and macrophages [92]. Both host humoral and cellular immune mechanisms were involved in the continued suppression of Friend erythroleukemia metastases after IFN-*α* treatment in mice [91]. In addition, effective adaptive immunotherapy was observed in a T-cell lymphoma model, after the injection of tumor-sensitized spleen cells and IFN-*α*. By using antibodies against different immune cell populations, it has been shown that CD4^+^ T lymphocytes and CD8^+^ T lymphocytes were the major effectors in the antitumor activities induced by IFN-*α* [93, 94]. 

### 4.2. Antitumor Mechanisms of IFN-*λ*

Although IFN-*α* and IFN-*λ* signal quite similarly (Figure 1), the mechanisms underlying the antitumor activity of IFN-*λ* may be qualitatively different from IFN-*α*. As previously described, we initially investigated whether type III IFNs also possessed antitumor activities utilizing a gene therapy approach in the B16 melanoma model. Since secreted IFN-*λ* did not affect the proliferation rate of B16 melanoma cells *in vitro*, studies in the B16 melanoma model suggested that IFN-*λ* acted through host mechanisms to elicit its antitumor activity [4]. However, we did not observe a significant long-lasting immunity, implying that there may be a lack of effective adaptive immunity in the mice which rejected the tumor. On the other hand, we noticed a reduction in tumor vascularity in the presence of IFN-*λ*, suggesting a potential role of IFN-*λ* in the tumor microenvironment [4]. Since we found that keratinocytes are highly sensitive to IFN-*λ* and they are known to interact with melanocytes, the cells from which the melanoma originates, we suggested that IFN-*λ* delivery to the tumor microenvironment may affect the function of the keratinocytes as well as other stroma cells thereby promoting inhibition of tumor growth [4]. NK cells, the major effectors of innate immunity, could also be recruited to the tumor microenvironment and help destroy the tumor cells. Two groups have reported that NK cells played a role in the antitumor mechanisms of IFN-*λ*. Sato et al. [68] have described the involvement of NK cells in melanoma and colon cancer antitumor responses. They have shown that transient transduction of B16 cells with mouse IFN-*λ* cDNA enhanced MHC class I and Fas expression, suppressed cell proliferation by inducing increased caspase-3/7 activity, increased p21^Waf1/Cip1^ levels, and dephosphorylated Rb (Ser^780^) *in vitro* [68]. This meant that IFN-*λ* was able to induce cell cycle arrest and apoptotic cell death *in vitro*. In addition, they have demonstrated that overexpression of IFN-*λ* inhibited local and pulmonary metastatic tumor formation *in vivo*. Depletion of NK cells, by injecting an anti-asialo GM1 antibody before tumor cells injection, revealed that NK cells are important in this IFN-*λ*-mediated tumor growth inhibition *in vivo*, suggesting that IFN-*λ* activated the innate immune response [68]. Numasaki et al. [69] have also implicated NK cells, polymorphonuclear neutrophils, and CD8^+^ T cells in the antitumoral activity are induced by IFN-*λ* in the MCA205 murine fibrosarcoma mouse model. Inoculation of MCA205-IFN-*λ* cells into mice enhanced IFN-*γ* production and cytotoxic T-cell activity in the spleen. The antitumor activity of IFN-*λ* was partially dependent on IFN-*γ*. In addition, IFN-*λ* increased the total number of splenic NK cells in severe combined immunodeficiency (SCID) mice, enhanced IL-12-induced IFN-*γ* production *in vivo, *and expanded spleen cells in C57BL/6 mice. Furthermore, they reported that IL-12 augmented the IFN-*λ*-mediated antitumor activity in the presence or absence of IFN-*γ*. Based on their findings, they suggested that IFN-*λ* is able to induce both innate and adaptive immune responses to suppress *in vivo* tumor growth [69].

Our recent study in the BNL hepatoma model also revealed that NK cells are implicated in the antitumor activity induced by IFN-*λ* and probably more potently than IFN-*α*. However, in contrast to IFN-*α*, we did not detect any response after *in vitro* treatment of NK cells by IFN-*λ*, suggesting that IFN-*λ* may activate other cells, which then mediate NK cell activation [45]. There was also a marked NK cell infiltration in IFN-*λ*-producing tumors. In addition, IFN-*λ* and, to a lesser extent, IFN-*α* enhanced immunocytotoxicity of splenocytes primed with irradiated BNL cells. Splenocyte cytotoxicity against BNL cells was dependent on IL-12 and IFN-*γ* and mediated by dendritic cells. In contrast to NK cells, isolated from spleen, CD11c^+^ and mPDCA^+^ dendritic cells responded directly to IFN-*λ*, suggesting that the effects of IFN-*λ* on NK cells are mediated by other IFN-*λ*-responsive cells, such as DCs [45]. On the other hand, a significant decrease in CD4^+^CD25^+^Foxp3^+^ Tregs was observed in mice inoculated with BNL cells secreting IFN-*α*, whereas the moderate decrease in Tregs observed in mice receiving BNL cells secreting IFN-*λ* was not statistically significant [45]. Therefore, antitumor mechanisms activated by IFN-*α* and IFN-*λ* may differ; IFN-*λ* increased the number of NK cells at the tumor site whereas IFN-*α* had a stronger effect on Tregs in the BNL model.

These studies altogether suggest that although IFN-*α* and IFN-*λ* signal quite similarly, differences exist in their biological potency, kinetics, and the sets of target cells sensitive to IFN-*λ* and IFN-*α*. Therefore, these two types of IFNs may have distinct physiological functions.

## 5. IFN-***λ*** and IFN-**α**: Allies in Achieving Higher Antitumor Activities?

Unlike IFN-*α*, only a small subset of cells are sensitive to IFN-*λ*, implying that its potential clinical use may be associated with limited side effects. This presumption raises the question whether IFN-*λ* could be an alternative to IFN-*α* in cancer therapy. However, despite the severe and numerous side effects inherent to IFN-*α* treatment [65], we believe that alternative treatment to IFN-*α* should be weighed first against the real benefits to patients in terms of overall survival and their tumor clearance. We have demonstrated in the BNL hepatoma model that the combination of IFN-*λ* and IFN-*α* could achieve a marked antitumor activity in comparison with the use of each IFN alone [62]. The benefits of the combination therapy of IFN-*λ* and IFN-*α* have been demonstrated both by using a gene therapy approach and by direct administration of IFNs to the mice bearing the tumors. The mice injected with BNL cells secreting both IFN-*λ* and IFN-*α* can completely reject the tumor, in contrast to the mice that only received the BNL-IFN-*λ* cells or the BNL-IFN-*α* cells. Furthermore, mice bearing established tumors and treated with exogenous IFN-*λ* and IFN-*α* showed a drastic tumor repression. This effect was observed when the IFNs were delivered locally and even at low doses. Therefore, we believe that IFN-*λ* is not simply acting like IFN-*α*, with reduced side effects, but can be combined with IFN-*α* to achieve efficient antitumor activity. Combination of IFN-*λ* with low doses of IFN-*α*, which are subtherapeutic but less toxic [67], may improve IFN therapy and benefit cancer patients. Combinational therapy of IFN-*λ* and IFN-*α* may achieve ultimate antitumor activity by inducing complementary mechanisms directly on the tumor cells or by indirectly modulating the tumor microenvironment, thereby leading to the stimulation of the immune response against the tumor and the inhibition of tumor angiogenesis. By acting with different intensities on the same targets, IFN-*λ* and IFN-*α* may generate a high level of synergy, leading to a potent antitumor activity.

## 6. Conclusions

Similarly to IFN-*α*, IFN-*λ* has been shown to play an important role in cancer and viral disease treatment. Although the two IFNs act through an identical signaling pathway in the cell, the pattern of their activity seems to be different *in vivo*, implying that IFN-*λ* and IFN-*α* are not redundant cytokines. By acting on some targets with different intensities, we believe that IFN-*λ* and IFN-*α* act in concert to better control tumor development *in vivo*. Therefore, to achieve better treatments for viral diseases or cancers, we believe that the development of a combination therapy rather than the use of each IFN alone will be more beneficial for the patients. The combination of IFNs with other cytokines, growth factors, or their antagonists could also be an important strategy for the improvement of the IFN therapy. Transforming growth factor-beta (TGF*β*) which plays a dual role in cancer, mediating tumor-suppresive activities at early stages and prooncogenic activities at later stages of tumor progression [95, 96], could represent one potentially important modulator or mediator of the IFN response. Understanding the potential crosstalks between IFN-*α*, IFN-*λ* and other cytokines or growth factors, such as TGF*β*, could be rewarding and lead to new preclinical studies in animal models and new clinical trials resulting in better cancer treatments.

## Figures and Tables

**Table 1:** Clinical indications of IFNs. IFN-*α* with different trade names is the most indicated in the clinic. IFN-*β* is mostly indicated for the treatment of relapsing remitting multiple sclerosis. IFN-*γ* is only indicated for the chronic granulomatous disease. IFN-*λ*, the new type of IFN, was tested for patients with chronic hepatitis C.

IFN type	Indications in the clinic
IFN-*α*	Hairy cell leukemia
	Multiple myeloma
	Chronic myeloid leukemia
	Follicular lymphoma
	Cutaneous T lymphoma
	Kaposi sarcoma
	Melanoma
	Renal cell carcinoma
	Hepatocellular carcinoma
	Condyloma accuminata
	Hepatitis B
	Hepatitis C

IFN-*β*	Multiple sclerosis

IFN-*γ*	Chronic granulomatous disease

IFN-*λ*	Hepatitis C
